# Suppression of lncRNA MALAT1 by betulinic acid inhibits hepatocellular carcinoma progression by targeting IAPs via miR‐22‐3p

**DOI:** 10.1002/ctm2.190

**Published:** 2020-10-14

**Authors:** Feiyu Chen, Zhangfeng Zhong, Hor Yue Tan, Wei Guo, Cheng Zhang, Chien‐Shan Cheng, Ning Wang, Junguo Ren, Yibin Feng

**Affiliations:** ^1^ School of Chinese Medicine, Li Ka Shing Faculty of Medicine The University of Hong Kong Hong Kong S.A.R. P. R. China; ^2^ Institute of Basic Medical Sciences, Xiyuan Hospital China Academy of Chinese Medical Sciences Beijing P. R. China

**Keywords:** apoptosis, autophagy, betulinic acid, cell death, hepatocellular carcinoma, lncRNA

## Abstract

Betulinic acid (BA) is a natural product extracted from a broad range of medicinal and edible herbal plants. Previous studies showed that BA induces cell death in tumors derived from multiple tissues; however, the underlying mechanism remains obscure. The present study aimed to study the effects of BA on autophagy and apoptosis of hepatocellular carcinoma (HCC). Human HCC cell lines and orthotopic HCC implanted mice were employed to examine the BA‐induced tumor suppression; RT^2^ long noncoding RNA (lncRNA) PCR array and database analysis were used to explore the possible mechanisms; validation of pathways was performed using siRNA and miRNA inhibitors. The results indicated that BA regulated autophagy and induced apoptosis in HCC. The degradation of inhibitor of apoptosis proteins (IAPs), the conversion of LC3‐I to LC3‐II, and p62 accumulation were enhanced by BA, thereby suggesting that the downregulation of IAPs and autophagic cell death are induced by BA. The addition of autophagy and lysosomal inhibitors indicated that BA induced autophagy‐independent apoptosis via degradation of IAPs. Moreover, RT^2^ lncRNA PCR array and database analysis suggested that BA downregulated the levels of lncRNA MALAT1, which is considered to be an oncogene. Further investigations demonstrated that lncRNA MALAT1 functioned as a ceRNA (competing endogenous RNA) to contribute to BA‐mediated degradation of IAPs by sponging miR‐22‐3p. Therefore, BA could be developed as a potential anticancer agent for HCC.

## Graphical abstract

Betulinic acid (BA) inhibited tumor growth of HCC in vivo and in vitro.

BA induced cell death through the inhibition of autophagic flux and autophagy‐independent apoptosis in HCC.

BA promoted apoptosis in HCC through MALAT1 inhibition in vivo and in vitro.

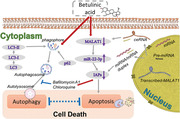

## 1 INTRODUCTION

Liver cancer is one of the most highly fatal cancers around the world.^1^ Among all the incidences of liver cancers, hepatocellular carcinoma (HCC) makes up 70‐90% of the whole liver cancers worldwide.^2^ The evasion of cell death represents a characteristic property of malignancies, and therefore, targeting cell death is one of the main strategies to combat cancers. Apoptosis and autophagy are two major types of programmed cell death. Extensive evidence has indicated the importance of modulating these two types of programmed cell death in human cancers.^3^

Herbal medicines have been used as food or supplements since ancient times.^4^, ^5^ A wealth of natural products originated from medicinal herbs have been shown to have therapeutic effects in many diseases such as cancer, heart failure, and malaria.^6^ Betulinic acid (BA) is a natural pentacyclic triterpene, and it can be derived from several Chinese herbal medicines such as *Zizyphus joazeiro* and *Prunella vulgaris*.^7^ It has been shown to have anticancer activity,^8^ and it is toxic to tumor cells but not to normal cells or tissues, which indicates its therapeutic potential for cancer treatment.^9^ BA has been shown to target the mitochondrial pathway to induce apoptosis in cancer cells.^8^, ^10^

In addition to apoptosis, autophagy, which is a physiological control process for the clearance of misfolded proteins and damaged organelles, is now considered a major form of cell death.^11^ It maintains cells homeostasis by limiting the toxic accumulation of impaired or superfluous proteins and organelles in order to sustain cell survival.^12^ In the process of autophagy, autophagosome, a double‐membrane vesicle, is formed and fused with lysosomes to form autolysosomes, whose contents can be degraded and digested by lysosomal enzymes.^13^ Moreover, it has been shown to take a dual role in cancers. Both survival‐supporting and death‐promoting roles of autophagy have been found in malignant transformation, suggesting its complex and paradoxical role in cancer.^14^ Inhibitor of apoptosis proteins (IAPs) are cell survival factors, which are involved in apoptotic and nonapoptotic cell death,^15^ and they have been found to be overexpressed in many cancers; therefore, targeting IAPs has been suggested as a therapeutic strategy for cancer treatment.^16^

Long noncoding RNAs (lncRNAs) have more than 200 nucleotides, having no protein‐coding capacity.^17^ By most estimates, human lncRNAs account for 98% of the human genome, which considerably exceeds the abundance of protein‐coding genes.^18^ An lncRNA, metastasis‐associated lung adenocarcinoma transcript 1 (MALAT1), has been reported to be a prognostic marker for poor clinical outcomes in nonsmall cell lung cancer patients.^19^ Its potential oncogenic role has been recently suggested in HCC.^20^, ^21^ Additionally, microRNAs (miRNAs) have been demonstrated to act as tumor oncogenes or suppressors in many cancers.^22^ A variety of HCC‐related lncRNAs, including MALAT1, have been shown to participate in cancinogenesis through binding with DNA, RNA, or proteins, or encoding small peptides.^23^, ^24^ They were demonstrated to act as sponges of miRNAs, leading to the dysregulation of miRNAs and subsequently the abnormal expression of corresponding target genes.^25^, ^26^, ^27^, ^28^

BA has been reported to induce cell death through apoptosis in a variety of cancer cells.^29^, ^30^ Nevertheless, the role of autophagy in BA‐induced cell death in HCC is unknown. In the current study, we investigated the anticancer effects of BA in HCC, with a focus on apoptosis and autophagy, and the potential mechanisms of action. Our results indicated that BA repressed the proliferation of HCC, PLC/PRF/5, and MHCC97L cells through apoptosis and autophagy. Likewise, tumor suppression by BA was also confirmed in a liver orthotopic transplantation mouse model. We showed that BA promoted the accumulation of autophagosomes and inhibited autophagic flux, thus suggesting that the anticancer effects of BA might be associated with autophagy. Further, we investigated the crosstalk between BA‐induced apoptosis and autophagy, two main types of programmed cell death, in HCC. Notably, MALAT1 has been reported to modulate expression of IAPs by acting as a proto‐oncogene.^31^ Here, we investigated the functional role and the mechanisms through which MALAT1 regulates BA‐induced apoptosis in HCC.

## 2 MATERIALS AND METHODS

### 2.1 Cell lines and cell culture

The two HCC cell line PLC/PRF/5 and MHCC97L, and the immortalized human liver cell line MIHA were kind gifts from Professor Tso Sai‐Wah (The University of Hong Kong, HKU), Dr Man Kwan (HKU), and Professor Guan Xinyuan (HKU), respectively. The cells were cultured in high glucose Dulbecco's Modified Eagle Medium (DMEM, Invitrogen, MA) supplemented with 10% fetal bovine serum (FBS, Invitrogen), and 10 mL/L of 100 antibiotic antimycotic solution (Sigma, MO). The cells were incubated at 37°C in 5% CO_2_ and 95% air.

### 2.2 Animal study

All animal assays were performed according to an approved protocol from the Committee on the Use of Live Animals in Teaching and Research (CULATR) from HKU. Orthotopic HCC‐implanted model was established in 5‐6 weeks old BALB/c nude mice according to our previous publications.^32^, ^33^ Briefly, a luciferase reporter was transfected into MHCC97L cells, which were then injected subcutaneously into the right flank region of the mice. The solid tumor generated was sliced into small pieces (around 1 × 1 mm) and then anchored to the liver surface of mice that underwent median laparotomy. After 1 week of observation, successfully transplanted mice were selected and divided into two groups (six mice/group), untreated and BA‐treated mice. The treated mice were administrated with BA (TRC, Toronto, Canada; 10 mg/kg/day, every 2 days) via intraperitoneal injection for 4 weeks. In the following study of MALAT1, siRNA for MALAT1 and negative control (NC) (Thermo Fisher Scientific, MA) were injected via caudel vein, according to the manual's instructions. The measurement of hepatic tumor growth was performed once a week using an in vivo imaging system. At the end of the treatment, all the mice were euthanized, and the liver and the tumor were dissected for further analysis.

### 2.3 Histology and immunoflurescence

Tissue samples were fixed with 10% formalin and dehydrated by various concentrations of alcohol, according to our previous study.^34^ After embedding in paraffin, tissues were cut into sections of approximately 5 μm. In the process of hematoxylin and eosin (H&E) staining, the slides were incubated in Mayer's hematoxylin and eosin Y solution (Sigma‐Aldrich), and then the images were taken using an optical microscope (Olympus, Tokyo, Japan). For immunohistochemical staining (IHC), the slides were incubated with antibodies against Ki‐67 (Cell Signaling Technology, MA) at 4°C overnight, and then incubated with secondary antibodies at 20‐25°C for 2 hours, followed by mounting with mounting medium (Dako, Glostrup, Denmark). Images were taken using an optical microscope (Olympus). For immunofluorescence analysis, the slides were incubated with antibodies against XIAP or survivin (Abclonal, MA) at 4°C overnight, and then incubated with secondary antibodies Alexa Fluor 488 (Invitrogen), Alexa Fluor 568 (Invitrogen) at 20‐25°C for 2 hours or DAPI (Invitrogen), followed by mounting with fluorescent mounting medium (Dako). Images were captured by using a confocal microscope (Carl Zeiss LSM 780; Jena, Germany).

### 2.4 MTT assay

MTT assay was employed to measure cell viability. Cells were seeded onto 96‐well plates (1 × 10^4^/well). On the next day, the cells were administrated with control 0.1% dimethylsulfoxide (DMSO; Sigma‐Aldrich), chloroquine (Sigma‐Aldrich), or different concentrations of BA, either alone or in combination. Following 24, 48, or 72 hours of incubation, MTT (thiazolyl blue tetrazolium blue; 1 mg/mL; Sigma‐Aldrich) was added to the cells. After incubating for 4 hours, the absorbance was measured at 570 nm.

### 2.5 2‐D wound healing assay

Cell migration was evaluated as previously described.^34^ Briefly, cells were plated onto 6‐well plates (1 × 10^5^/well). After a monolayer‐confluent cell layer formed, a sterile micropipette tip (200 μL) was used to create a wound (approximately 0.5 mm), followed by gently rinsing with PBS twice. Then, the cells were subjected to the indicated dosages of BA for 48 hours. The images were captured every 24 hours, over a period of 48 hours, under an inverted microscope (Olympus). The wound width between the edges of the cell‐free area was measured by Image‐J software (NIH, MD).

### 2.6 3‐D transwell assay

Cells were plated onto the upper chamber of transwell (5 × 10^4^/well; 8 μm; BD, NJ) with matrigel. DMEM containing 10% FBS was added to the bottom chamber. Twenty‐four hours later, the cells in the top chamber were gently removed. Subsequently, the invading cells to the bottom chamber were fixed with 4% paraformaldehyde overnight, followed by 2 hours of staining with 0.1% crystal violet solution. Images were obtained using an optical microscope. The number of invading cells was measured by manually counting in three randomly selected fields.

### 2.7 Colony formation assay

Colony formation ability was evaluated as previously described.^35^ In brief, cells were plated onto 6‐well plates (1000/well) and allowed to attach, and then indicated dosages of BA were added to the cells. After 10 days of incubation, the cells were gently rinsed with PBS, and colonies were fixed for 2 hours in 4% paraformaldehyde. Colonies were washed once again, followed by 30 minutes of staining with 0.1% crystal violet solution. Images were obtained by an inverted microscope and manually counted in three randomly selected fields.

### 2.8 Flow cytometry

Cells were plated onto 6‐well plates (1 × 10^5^/well) and exposed to BA for 48 hours. For detection of apoptosis, all the cells were harvested and processed with FITC annexin V‐PE (FITC)/7‐AAD Apoptosis Detection kit (BD) following the manufacturer's manuals. The stained cells were rinsed with PBS once and analyzed using a flow cytometer (BD). For analysis of cell cycle, the cells were collected and fixed in 70% ethanol overnight at 4°C. Next, the cells were stained with PI for 15 minutes at 20‐25°C in the dark and acquired with the flow cytometer.

### 2.9 Western blot

The protein was extracted from liver tissues and cell harvests. Equal amounts of extracted protein (approximately 20 μg) were separated by using SDS‐PAGE gel and transferred onto polyvinylidene fluoride membrane (Bio‐Rad, CA). After 2 hours of blocking with 5% BSA, the membranes were incubated with primary antibodies (GAPDH, LC3, p62, cIAP1, cIAP2, XIAP, and survivin) at 4°C overnight, followed by incubation with secondary antibodies at 20‐25°C for 2 hours. Antibodies against GAPDH, cIAP1, cIAP2, LC3, and p62 were obtained from CST, while antibodies against XIAP and survivin were obtained from Abclonal. The immunoblots were visualized using electrochemiluminescent reagents (GE Healthcare, IL) following the manufacturer's instructions, and quantified using Quantity One System image analyzer (Bio‐Rad).

### 2.10 Quantitative real‐time polymerase chain reaction

The RNA of cells and tissues were extracted using Trizol (Takara, Kyoto, Japan). mRNA was converted to cDNA using PrimeScript RT master mix (Takara). Quantitative real‐time polymerase chain reaction was performed by SYBR Green master mix (Takara) using LightCycler 480 System (Roche, Basel, Switzerland).^36^ The human GAPDH was applied as an internal reference. The primers for miR‐22‐3p and U6 were purchased from Qiagen (MD). The other primers used are shown in Table 1.

**TABLE 1:** Quantitative PCR primers and corresponding sequences

Gene list	Forward sequence	Reserve sequence
GAPDH	GAGCCCGCAGCCTCCCGCTT	CCCGCGGCCATCACGCCACAG
LC3B	AGCAGCATCCAACCAAAATC	CTGTGTCCGTTCACCAACAG
P62	TGCCCAGACTACGACTTGTG	AGTGTCCGTGTTTCACCTTCC
cIAP1	AGCTAGTCTGGGATCCACCTC	GGGGTTAGTCCTCGATGAAG
cIAP2	GGAAATTGACCCTGCGTTATACAGA	TCTCGGTCCATACACACTTTACACATT
XIAP	CCCAAATTGCAGATTTATCAACG	TGCATGTGTCTCAGATGGCC
Survivin	AGAACTGGCCCTTCTTGGAGG	CTTTTTATGTTCCTCTATGGGGTC
MALAT1	GACGGAGGTTGAGATGAAGC	ATTCGGGGCTCTGTAGTCCT

### 2.11 Transmission electron microscopy

MHCC97L cells were digested and centrifuged for 5 minutes at 800 rpm. The cell pellet was processed and prepared by the Electron Microscope Unit at HKU. The cells were subsequently visualized and photographed with a transmission electron microscope (Philips LMSI CM100, Eindhoven, The Netherlands).

### 2.12 Monodansylcadaverine staining

Cells were plated onto 6‐well plates (5 × 10^4^/well) and treated with BA for 24 h, followed by staining with monodansylcadaverine (MDC) (0.05 mM) at 37°C for 10 minutes. At last, the images were captured by a fluorescence microscope (Carl Zeiss LSM 780).

### 2.13 Quantification of GFP‐LC3 puncta

Cells were plated onto 6‐well plates (5 × 10^4^/well), and infected with adenovirus expressing mCherry‐GFP‐LC3 fusion proteins (Addgene, MA). Then, the specified concentrations of BA were added to the cells. Twenty‐four hours later, the cells were monitored by a fluorescence microscope (Carl Zeiss LSM 780).

### 2.14 RT^2^ lncRNA PCR array

MHCC97L cells were plated onto 6‐well plates (1 × 10^5^/well) and administrated without or with BA for 48 hours, and total lncRNAs were extracted using RT^2^ first strand kit (Qiagen). Next, the RNAs were transferred to the plate of RT^2^ lncRNA PCR array (Qiagen) as per the manual's instructions. The signals were detected using LightCycler 480 System (Roche).

### 2.15 Luciferase reporter assay

The fragment of MALAT1 containing the miR‐22‐3p binding sites and the corresponding mutants were subcloned into the pmirGLO dual‐luciferase miRNA target expression vector (Promega, WI) to construct MALAT1‐mutated‐type (MALAT1‐MUT) and MALAT1‐wild‐type (MALAT1‐WT) (GenePharma, Shanghai, China). HCC cells (PLC/PRF/5 and MHCC97L cells) were plated onto 24‐well plates (2 × 10^6^/well). Twenty‐four hours later, the plasmids MALAT1‐WT or MALAT1‐MUT were co‐transfected into HCC cells along with miR‐NC or miR‐22‐3p mimics using Lipofectamine 3000 transfection reagent (Life Technologies, MA). Relative luciferase activities were measured after transfection of 48 hours according to the manufacturer's manuals of the Dual‐Luciferase Reporter Assay System kit (Promega).

### 2.16 Statistical analysis

All the assays were carried out for three times, and the results are presented as mean ± SD or mean ± SEM for each set of experiments. Statistical significance was analyzed using two‐way ANOVA or student's t‐test. *P*‐value < .05 was considered as statistically significant.

## 3 RESULTS

### 3.1 BA inhibited tumor growth of HCC in vivo and in vitro

**FIGURE 1:**
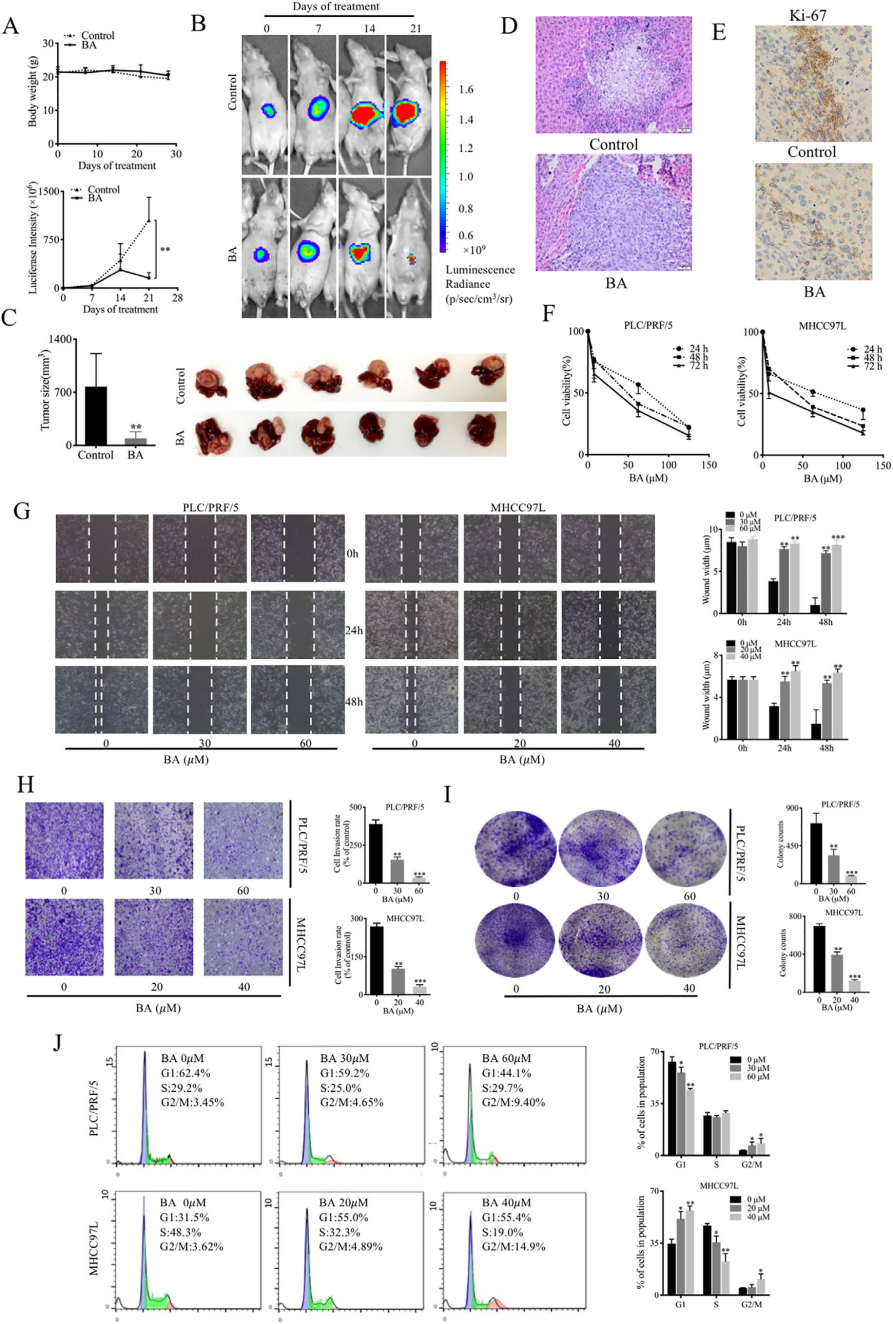
BA inhibited tumor growth of HCC in vivo and in vitro. (**A**), Body weight across 28 days of treatment in mice. The body weight was measured everyday. (**B**), Representative fluorescence images and graphs of orthotopic HCC implanted mice in response to BA (10 mg/kg/day every 2 days). The tumor growth was monitored using luciferase in vivo imaging system every week. (**C**), Tumor size and representative images of livers collected from orthotopic HCC‐bearing mice with or without BA treatment. The hepatic tumor size was compared between control and treatment groups. ^**^ *P *< .01, compared to control group. (**D**), Hematoxylin and eosin (H&E) staining was performed in hepatic sections of control and BA‐treated mice. (**E**), Immunohitochemical staining (IHC) was performed to observe the Ki67 expression in hepatic sections of control and BA‐treated mice. (**F**), PLC/PRF/5 and MHCC97L cells were treated with indicated concentrations of BA for 24‐72 hours. The cell viability was performed using MTT assay. (**G**), Wound healing monitored in HCC cells treated with indicated concentrations of BA for 24‐48 hours. The healing ability was observed on an inverted microscope after 24 and 48 hours, and the wound width was measured by Image‐J software. (**H**), Transwell invasion assay was performed in HCC cells after 48 hours of BA treatment. The cell invasion rate was counted manually by the number of cells that invaded onto the bottom membrane in three random fields. (**I**), Colony formation in response to BA. The HCC cells were treated with indicated concentrations of BA for 10 days. The colony formation ability was quantified by counting the number of spheres per field. ^**^ *P *< .01, ^***^ *P *< .001, compared to control group. (**J**), Cell cycle distribution following BA treatment. The cell cycle was analyzed by flow cytometry after treatment with indicated concentrations of BA for 48 hours

To examine the anticancer effects of BA in HCC in vivo, we established an orthotopic HCC mouse model in BALB/c athymic mice that resembles human HCC. Body weight was measured everyday, and no difference was observed after BA treatment (10 mg/kg/day, every 2 days), indicating that BA has no toxicity in vivo (Figure 1A). Luciferase signal, monitored by an in vivo imaging system, showed that BA restrained the growth of orthotopic HCC tumors, while the luciferase intensity in untreated mice was elevated continuously throughout 21 days (Figure 1B). Tumor size measurement also revealed that BA treatment restricted tumor growth in mice (Figure 1C). Taken together, these findings suggested that BA suppressed tumor proliferation and growth in HCC implanted mice. As invasion is a critical indicator of tumor progression, we also examined the role of BA in metastasis in vivo. H&E staining and IHC were used to visualize the hepatic tumor tissues. The BA‐treated group had a well‐defined layer of hepatic tumor tissues without notable invasion; however, notable invasion was observed in the hepatic tumor tissues of untreated mice (Figure 1D). This confirmed the inhibitory effect of BA on HCC cell invasion. Similarly, suppression of Ki67 expression could be observed in BA‐treated group (Figure 1E). Our findings suggested that BA exerted potent inhibitory effects on HCC tumor growth and cell invasion in vivo.

The effect of BA on cell viability was assessed by the MTT assay in HCC cells, PLC/PRF/5, and MHCC97L. BA demonstrated anticancer effects on cell viability in a dose‐dependent fashion in vitro (Figure 1F). The IC_50_ values of BA were 133.3, 63.04, and 23.93 μM at 24, 48, and 72 hours in PLC/PRF/5 cells, respectively, whereas they were 77.35, 40.02, and 9.47 μM at 24, 48, and 72 hours in MHCC97L cells, respectively. The 2‐D wound healing assay was also exploited to detect the inhibitory effects of BA on migration and cell proliferation, which were measured by wound closure. The wound closure was monitored over a period of 48 hours, and the results showed that the untreated cells migrated into the scratched space after 24 and 48 hours, but the wound was still open after BA treatment (Figure 1G). Higher concentrations of BA had a higher impact on the wound closure, and it completely prevented cell movement at 60 μM after 48 hours of treatment, thus illustrating the inhibitory effect of BA on the migration ability of HCC cells.

As cell invasion is one of the indicators of tumor progression, the 3‐D transwell assay was performed to study whether BA could also block cell invasion in vitro. As observed, there was a smaller proportion of cells that passed through the chamber membrane in the BA‐treated group in comparison to that in the untreated cells, and the cell invasion rate was decreased in a dose‐dependent manner (Figure 1H). Consistent with this, we also found that there were fewer colonies in BA‐treated cells compared to those in untreated cells, after 10 days of culture, indicating that the repressive effect of BA on cell proliferation was long term in HCC cells (Figure 1I). In addition, the cell cycle was also studied to determine the cell population in different phases of the cell cycles. This analysis showed that BA increased the proportion HCC cells at the G2/M phase (Figure 1J). Collectively, these findings suggested that BA induced cell arrest at the phase of G2/M as well as inhibited cell migration and invasion, as well as colony formation in HCC cells.

### 3.2 BA induced cell death through the inhibition of autophagic flux in HCC

**FIGURE 2:**
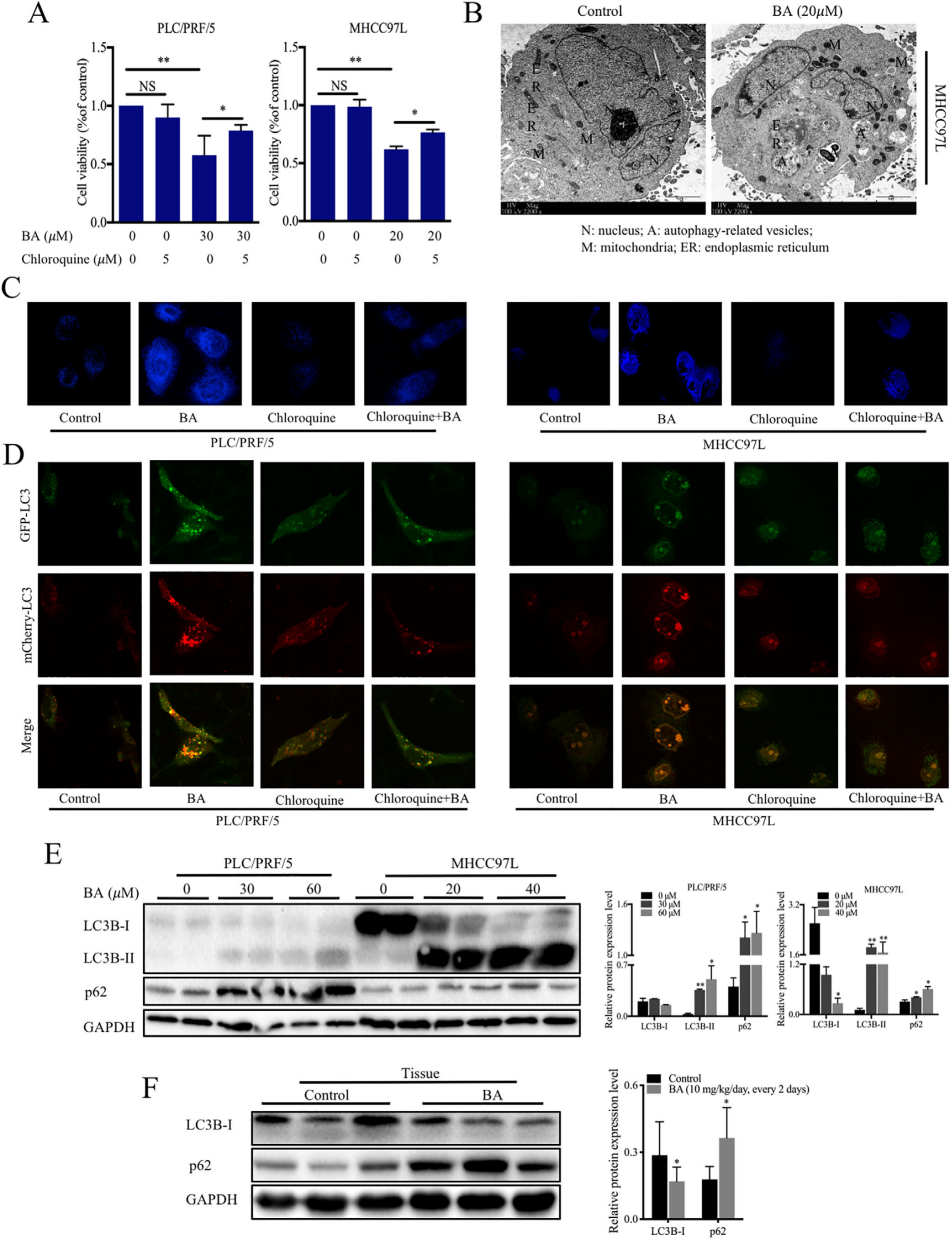
BA induced cell death through the inhibition of autophagic flux in HCC. (**A**), The cell death was assessed by MTT assay following BA treatment for 48 hours in the absence or presence of autophagy inhibitor, chloroquine, in HCC cells. (**B**), Transmission electron microscopy (TEM) images of MHCC97L cells with or without BA administration for 48 hours. (**C**), Monodansylcadaverine (MDC) staining in response to BA. The cells were visualized using a fluorescence microscope. (**D**), Immunfluorescence images of mCherry‐GFP‐LC3‐expressed HCC cells. The cells were transfected with mCherry‐GFP‐LC3 vector and then were exposed to the indicated concentrations of BA or chloroquine for 48 hours. ^*^ *P *< .05 and ^**^ *P *< .01. (**E**), Representative immunoblots and graphs showing the protein levels of LC3 and p62 in lysates derived from cells treated with indicated concentrations of BA

Dysregulation of autophagy plays a critical part in cancer cell initiation, proliferation, metastasis, and expansion. To evaluate the role of autophagy in the anticancer activity of BA in HCC cells, chloroquine was employed as an autophagy inhibitor (Figure 2A). In the presence of chloroquine, there was a significantly less BA‐induced cell death compared to that using treatment with BA alone, thereby suggesting that autophagy plays a critical part in BA‐induced cell death in HCC cells. To further explore the involvement of autophagy in response to BA treatment, transmission electron microscopy was carried out to visualize autophagic vacuoles and apoptotic bodies in MHCC97L cells (Figure 2B). The untreated cells had normal nuclear and mitochondrial morphology, as well as fewer autophagosomes. However, BA‐treated cells had more autophagic vacuoles and apoptotic bodies, demonstrating the induction of autophagy by BA in HCC cells. Consistent with this, an increase in MDC staining was also observed in BA‐treated HCC cells (Figure 2C).

To further confirm the impact of BA on autophagy, we generated cells expressing mCherry‐GFP‐LC3 to simultaneously monitor the formation of autolysosomes as well as degradation events.^37^ The red puncta (mCherry positive) represent autolysosome formation, while the green puncta (GFP positive) represent autolysosome degradation. After incubation with 40 or 60 μM BA for 24 h, most of the puncta showed both green and red fluorescence, and these puncta became yellow when the images were merged, indicating that the autophagic flux was inhibited. Treatment of cells with chloroquine alone also resulted in an accumulation of green and red puncta (Figure 2D). Collectively, these findings indicated that BA repressed autophagic flux in HCC cells.

Microtubule‐associated protein 1 light chain 3 (LC3) is a well‐known monitor for autophagy in cancer cells,^38^ and p62 is an important LC3 interactor in autophagy.^39^ The results showed that LC3‐I was converted to LC3‐II (isoform II of light chain) upon BA treatment, and p62 protein and mRNA levels were upregulated (Figure 2E and F), indicating that it induced autophagy but the downstream autophagy pathway was blocked, and thus autophagic flux was inhibited in HCC. However, BA did not impair autophagic flux at a similar concentration in normal hepatocytes (data not shown). Taken together, these results suggested that BA increased cell death via the inhibition of autophagic flux in HCC.

### 3.3 BA induced cell death through autophagy‐independent apoptosis in HCC cells

**FIGURE 3:**
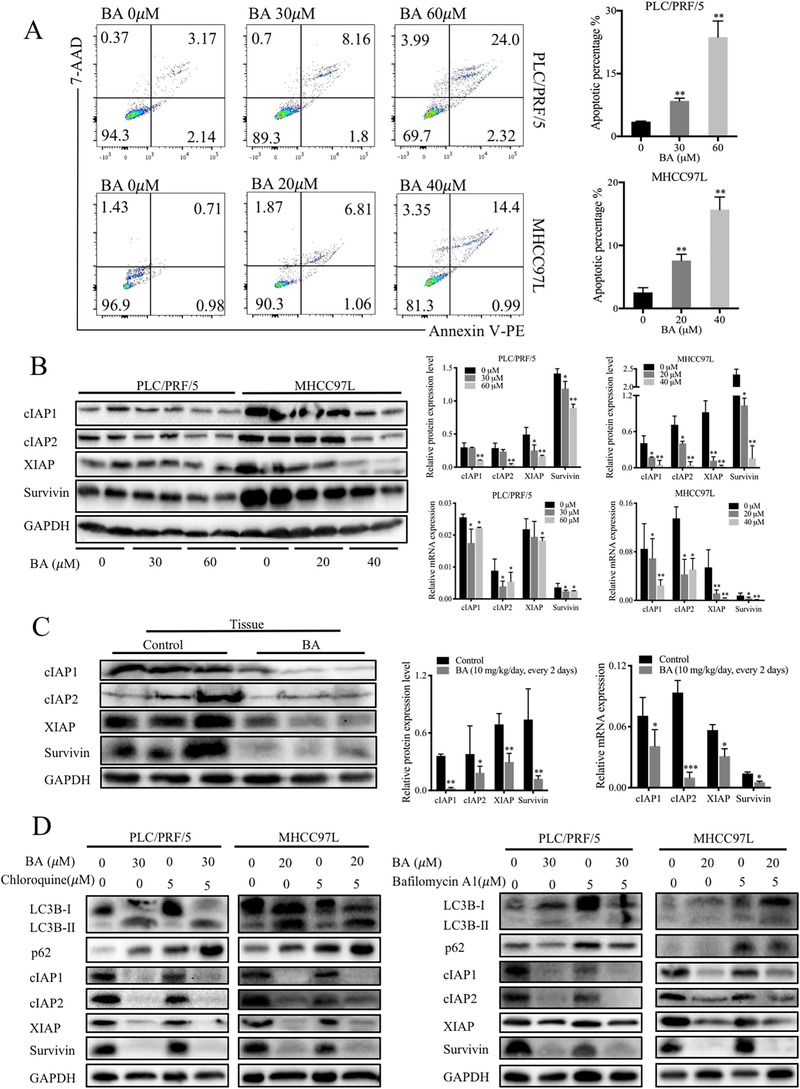
BA induced apoptosis through IAPs downregulation and inhibited autophagic flux that was independent of IAPs. (**A**), The cell apoptosis was assessed using annexin V‐PE/7‐AAD staining by flow cytometry after exposure to BA for 48 hours. Quantitative measurement of the rates in each phase of the apoptosis was conducted. (**B**) and (**C**), Representative immunoblots and graphs showing the protein and mRNA levels of inhibitor of apoptosis proteins (IAPs) in HCC cells and tumor tissues in response to BA. ^*^ *P *< .05 and ^**^ *P *< .01, compared with control group. (**D**), Representative immunoblots showing the protein levels of LC3, p62, and IAPs by BA in the presence of chloroquine or bafilomycin A1. ^*^ *P *< .05 and ^**^ *P *< .01, compared to control group

Previous studies have reported the anticancer activities of BA in cancer^8^; however, whether BA drives the apoptotic pathway involving IAPs remains undetermined. In this study, annexin V‐PE/7‐AAD double staining was employed to study the effects of BA on different stages of apoptosis. The results indicated that BA significantly increased the apoptosis in PLC/PRF/5 and MHCC97L cells (Figure 3A). In addition, we also examined the expression of IAPs, which are key regulators of apoptosis. The data showed that both protein and mRNA levels of cIAP1, cIAP2, XIAP, and survivin were significantly decreased following BA treatment (Figure 3B). Similarly, BA also decreased the protein and mRNA levels of cIAP1, cIAP2, XIAP, and survivin in mouse hepatic tissues (Figure 3C). These findings suggested that BA induced apoptosis by inhibiting the expression of IAPs in HCC cells.

To further investigate whether the IAPs pathway could regulate autophagy, expression of IAPs was evaluated in the presence of chloroquine (autophagy inhibitor). Immunoblotting results, however, showed that chloroquine did not induce any change in the expression of IAPs in presence of BA (Figure 3D), suggesting that IAPs were not involved in BA‐mediated autophagic changes. Meanwhile, the addition of chloroquine further confirmed that BA induced inhibition of autophagic flux.

### 3.4 BA inhibited the gene expression of MALAT1 in vivo and in vitro

**FIGURE 4:**
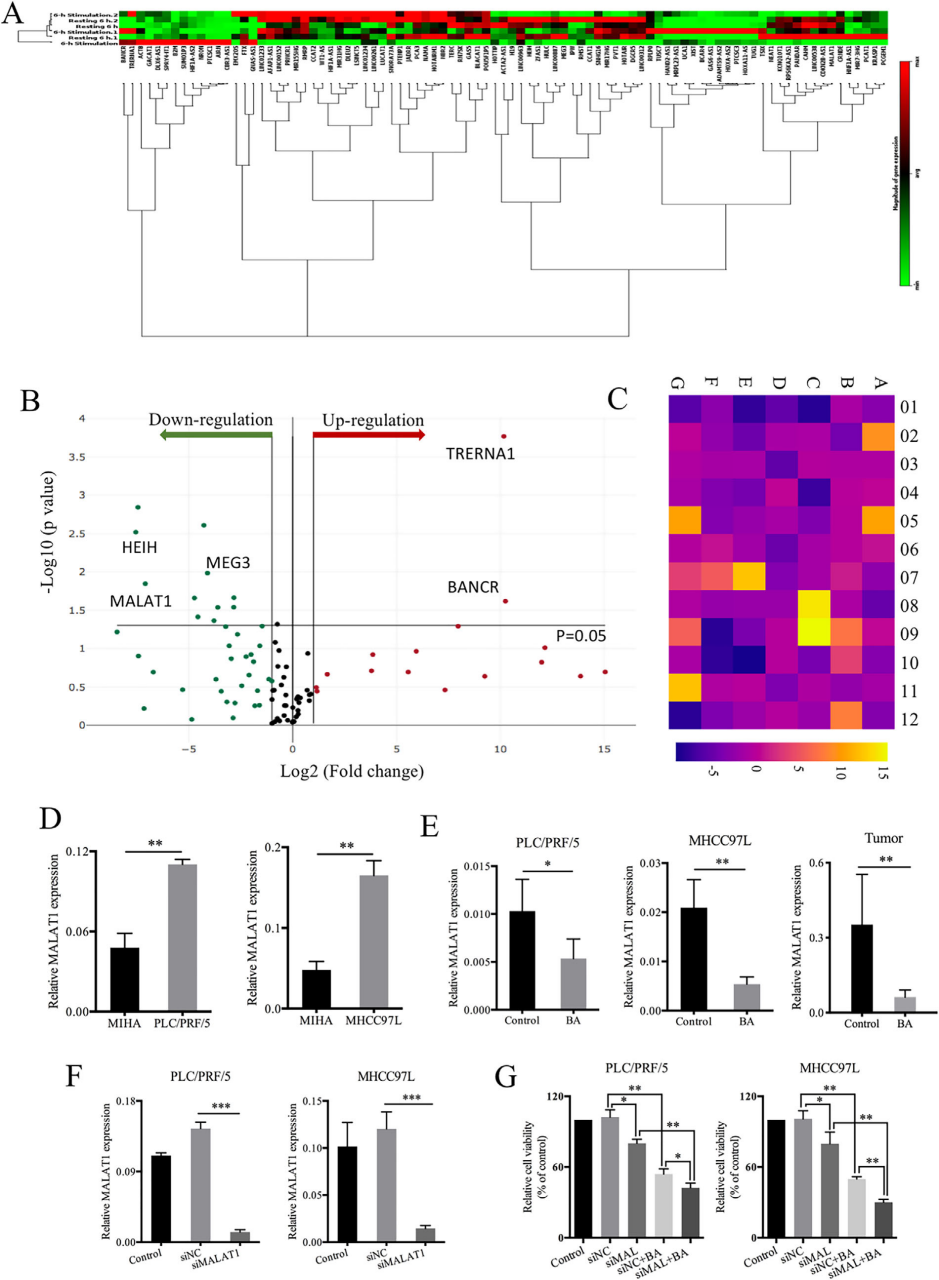
BA inhibited the gene expression of MALAT1 in vivo and in vitro. (**A**), MHCC97L cells treated with control or BA were subjected to RT^2^ lncRNA PCR array. The clustergram shows the downregulated and upregulated genes in responding to BA in MHCC97L cells. (**B**), The volcano plots show the downregulated and upregulated genes in responding to BA in MHCC97L cells. (**C**), The heatmap shows the changes of each oncogene of each well. Of 84 well, E01 represents MALAT1. (**D**), The MALAT1 expression was detected in both MIHA cells and HCC cells. ^*^ *P *< .05 and ^**^ *P *< .01, compared to control group. (**E**), The MALAT1 expression was detected in HCC cells and tumor tissue after exposure to BA. (**F**), siRNA of MALAT1 was used to establish the models in HCC cells. ^*^ *P *< .05 and ^**^ *P *< .01, compared to control group. (**G**), The effect of MALAT1 on cell proliferation was conducted with or without BA treatment using MTT assay. ^*^ *P *< .05 and ^**^ *P *< .01, compared to control group

The oncogenic role of MALAT1 in HCC has been recently suggested.^20^, ^21^ To investigate the potential role of lncRNA in HCC after BA treatment, RT^2^ lncRNA PCR array was applied in MHCC97L with or without BA treatment. It was observed that the expression of a series of genes was altered in respond to BA administration (Figure 4A); many genes were upregulated or downregulated in BA‐treated MHCC97L cells (Figure 4B). MALAT1 was downregulated significantly after BA treatment (Figure 4C). To confirm the overexpression of MALAT1 in HCC, RNA expression was examined in both MIHA cells and HCC cells. The results showed that the MALAT1 expression was significantly higher in PLC/PRF/5 and MHCC97L cells in comparison to that in MIHA cells (Figure 4D). In addition, PCR assays revealed that MALAT1 gene expression was downregulated by BA both in HCC cells and tumor tissue (Figure 4E). siRNA specific for MALAT1 was used to downregulate MALAT1 expression in HCC cells (Figure 4F). Then, the MTT assay was exploited to study the effect of MALAT1 on cell viability after BA treatment. The results showed that knockdown of MALAT1 reduced cell viability, and increased the BA‐induced inhibitory effect in HCC cells (Figure 4G). Collectively, these findings indicated that BA inhibited the expression of MALAT1 in vitro and in vivo.

### 3.5 BA promoted apoptosis in HCC through MALAT1 inhibition

**FIGURE 5:**
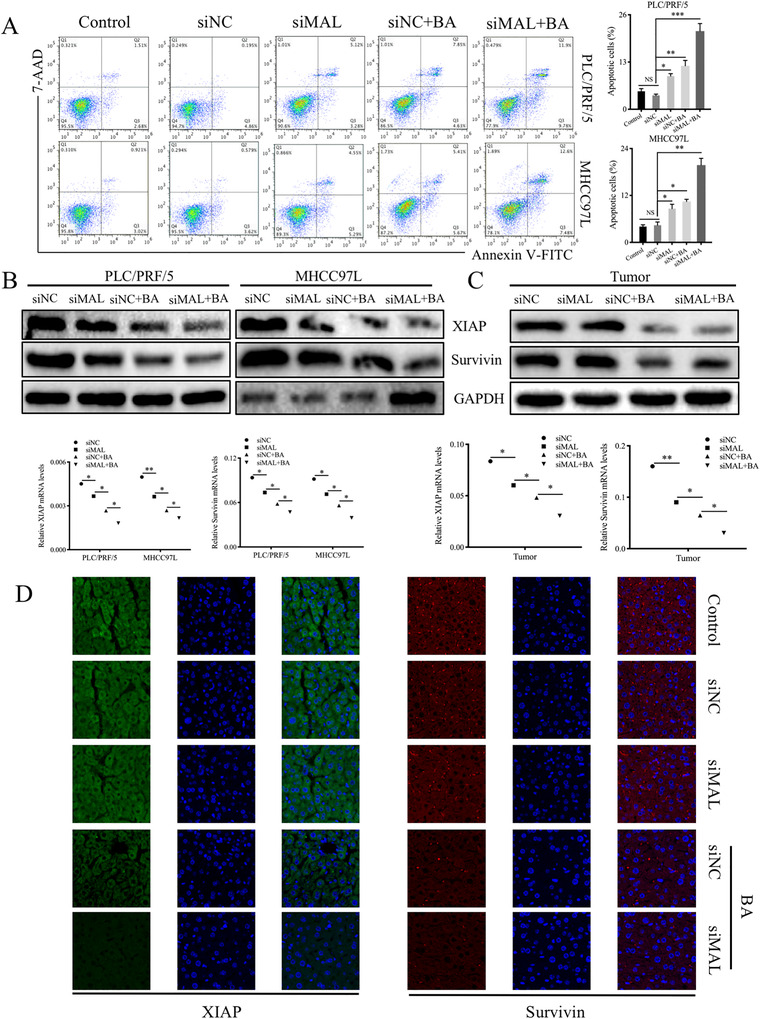
BA promoted apoptosis in HCC through inhibiting MALAT1. (**A**), Annexin V‐FITC/7‐AAD double staining was used to study the effect of MALAT1 and BA on cell apoptosis. (**B**), The expressions of XIAP and survivin in HCC cells were examined at both protein and RNA levels. ^*^ *P *< .05 and ^**^ *P *< .01, compared to control group. (**C**), The expressions of XIAP and survivin in mouse hepatic tumor tissues were examined. ^*^ *P *< .05 and ^**^ *P *< .01, compared to control group. (**D**), Immunofluorescence staining was used in tumor tissues to further confirm the role of MALAT1 in HCC growth as well as in BA‐induced HCC inhibition

Based on the inhibitory effect of BA on IAPs expression, we anticipated the association between IAPs and MALAT1 in HCC. To investigate the association of MALAT1 and cell apoptosis, annexin V‐FITC/7‐AAD double staining was exploited. This results showed that BA increased apoptosis of MALAT1 knockdown HCC cells (Figure 5A). The expressions of XIAP and survivin in HCC cells were examined, and the results indicated that at both protein and RNA levels, the expressions of XIAP and survivin were both downregulated (Figure 5B). Similar results were also observed in mouse hepatic tumor tissues (Figure 5C). To further confirm the role of MALAT1 in HCC growth as well as in BA‐induced HCC inhibition, immunofluorescence staining was performed in tumor tissues, and the results indicated that XIAP and survivin were downregulated when MALAT1 was knocked down (Figure 5D). Collectively, these results suggested that BA promoted apoptosis in HCC by inhibiting the expression of MALAT1.

### 3.6 MALAT1 functioned as a miR‐22‐3p sponge to contribute to BA‐induced apoptosis

**FIGURE 6:**
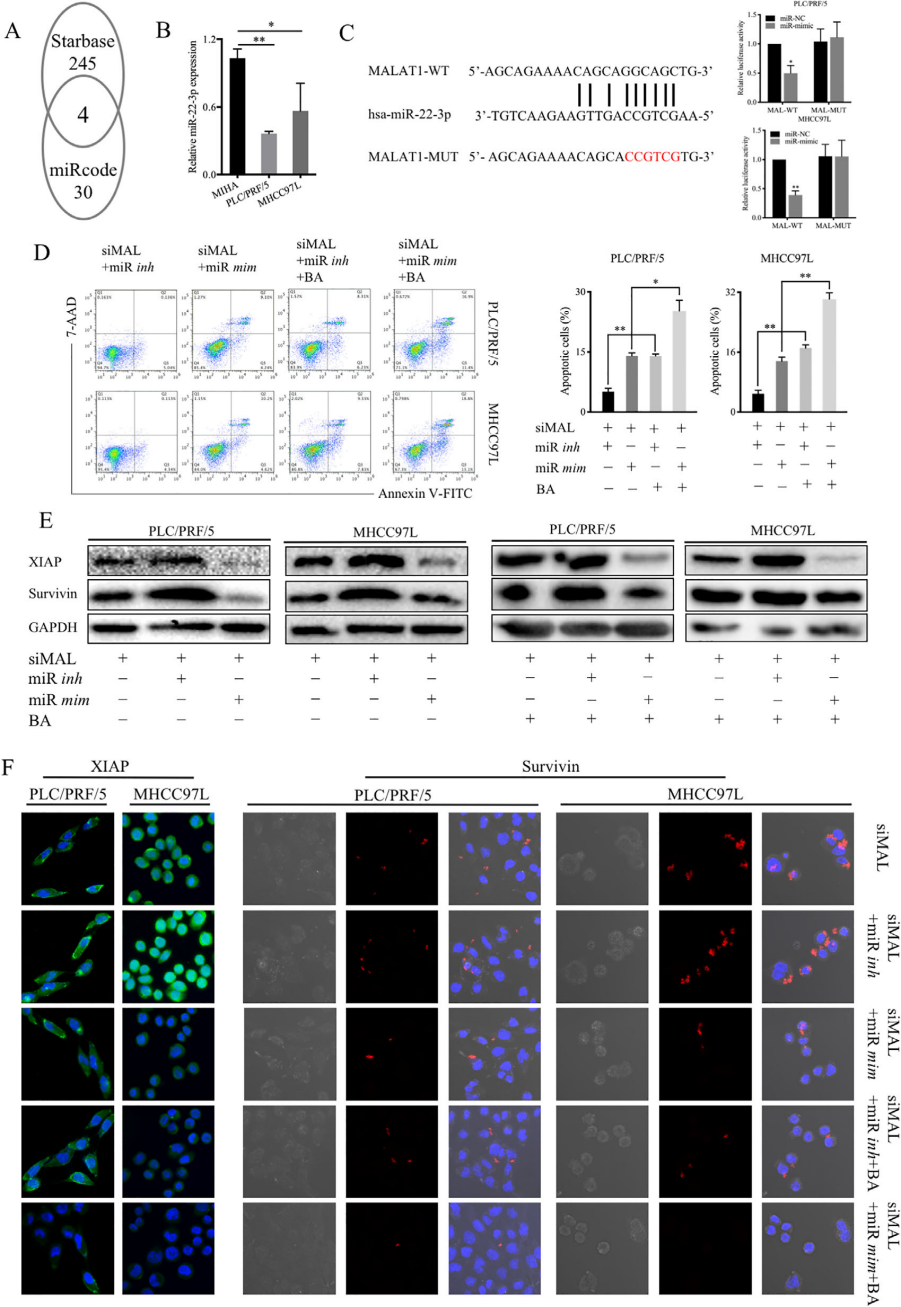
MALAT1 functioned as a ceRNA to contribute to BA‐induced apoptosis by sponging miR‐22‐3p. (**A**), A comprehensive search from public database Starbase and miRcode to investigate the relationship between MALAT1 and IAPs in HCC, four miRNAs including miR‐22‐3p, miR‐101‐3p, miR‐338‐3p, miR‐455‐5p were identified to link to MALAT1 in cancer, and XIAP is a target gene of miR‐22‐3p. (**B**), The expression of miR‐22‐3p was measured in normal liver cells and HCC cells using PCR. ^*^ *P *< .05 and ^**^ *P *< .01, compared to control group. (**C**), Dual luciferase assay in HCC cells was conducted to confirm the MALAT1/miR‐22‐3p axis in HCC progression. (**D**), Annexin V‐FITC/7‐AAD double staining was conducted to investigate the effect of the pathway MALAT1/miR‐22‐3p in HCC apoptosis after BA treatment. (**E**), The results were confirmed by immunoblotting. (**F**), the immunofluorescence staining was conducted to further confirm the pathway of MALAT1/miR‐22‐3p/IAPs

To further investigate the relationship between MALAT1 and IAPs in HCC, we conducted a comprehensive search in the public database Starbase and miRcode, and four miRNAs (miR‐22‐3p, miR‐455‐5p, miR‐338‐3p and miR‐101‐3p) were identified to be linked with MALAT1 in cancer. Further search using miRcode showed that XIAP is a target gene of miR‐22‐3p (Figure 6A). The expression of miR‐22‐3p was measured in normal liver cells and HCC cells using PCR, the results showed that miR‐22‐3p levels were lower in PLC/PRF/5 and MHCC97L cells compared to those in MIHA cells (Figure 6B). Thus, we hypothesized that MALAT1/miR‐22‐3p/XIAP could be the potential molecular pathway in HCC progression. In brief, MALAT1 can act as a ceRNA of miR‐22‐3p, and BA may affect the expression of IAPs by targeting MALAT1 through the activation of miR‐22‐3p. We verified this hypothesis using dual luciferase assay in HCC cells (Figure 6C). siRNAs for MALAT1, miR‐22‐3p inhibitors, and mimics were used to explore the mechanism of the anticancer activity of BA by annexin V‐FITC/7‐AAD double staining. The results showed that miR‐22‐3p inhibitors suppressed the apoptosis of HCC cells, whereas miR‐22‐3p mimics could promote the apoptosis even when MALAT1 was knocked down. Meanwhile, the administration of BA increased the apoptosis in miR‐22‐3p mimics‐treated cells in comparison to that in miR‐22‐3p inhibitors‐treated cells (Figure 6D). The immunoblotting results were consistent with the above results (Figure 6E). Moreover, the XIAP and survivin protein expression was examined using immunofluorescence staining of HCC cells treated in the same ways (Figure 6F). Collectively, these results showed that MALAT1 acted as a ceRNA of miR‐22‐3p, and BA affected the IAPs expression by targeting MALAT1 through the activation of miR‐22‐3p.

## 4 DISCUSSION

HCC is one of the main types of liver cancer worldwide. According to GLOBOCAN 2012, eastern Africa, sub‐Saharan western, Southeast Asia, and China exhibit the highest mortality and incidence of HCC.^40^ To date, effective agents are still needed to inhibit HCC progression. Apoptosis is known as a major form of programmed cell death, its aberrant regulation has a vital role in cancer initiation. Autophagy is regarded as another major type of programmed cell death,^11^ and many studies have suggested that the crosstalk between autophagy and apoptosis could control cell death and cell survival.^41^, ^42^ Defects in the physiological process of apoptosis and autophagy contribute to cell immortality, ultimately leading to tumor occurrence and expansion, so targeting apoptosis and autophagy for cancer treatment is reasonable.^41^, ^42^ BA has been reported to increase apoptosis in many cancers,^29^, ^30^ however, the role of autophagy in BA‐induced HCC cell death is unclear. Therefore, the current study evaluated the anticancer activity of BA in HCC in vivo and in vitro, with a focus on apoptosis and autophagy. We demonstrated that BA markedly inhibited HCC growth in vivo and in vitro, and induced apoptosis and autophagy to mediate cell death of HCC, PLC/PRF/5, and MHCC97L cells. Interestingly, BA induced autophagy but inhibited autophagic flux, leading to the accumulation of damaged organelles and misfolded proteins, and eventually cell death. Taken together, these results suggested that BA increased cell death through autophagy and apoptosis in HCC.

BA is a pentacyclic triterpene, which can be extracted from a variety of Chinese herbal medicines. It has desirable anticancer effects and is nontoxic to normal cells.^9^ Due to these attractive properties, native Americans started to use it as a folk remedy,^8^ and the National Cancer Institute has selected it as a candidate for the natural product‐based agents in cancer treatment.^43^ BA has many pharmacological activities, including tumor suppression and anti‐inflammatory effects.^29^, ^30^, ^44^ It has been showed to induce apoptosis and suppress metastasis in HCC cells and HepG2 xenograft mice.^45^ Consistent with this, we demonstrated that BA inhibited tumor progression and invasion in orthotopic HCC mice.

The antitumor property of BA has been shown to be associated with apoptotic cell death.^8^, ^29^, ^30^ Similarly, our results demonstrated that BA induced cell apoptosis, regulated cell cycle, and repressed cell invasion and colony formation in HCC, PLC/PRF/5, and MHCC97L cells. The IAPs are a group of proteins that serve as apoptotic suppressors, which inhibit cell death.^46^ Overactivation of IAPs renders the cells unable to undergo programmed cell death, thus they become resistant to chemotherapies and radiation therapies, so IAPs antagonists have been developed to treat cancer.^46^ Although BA‐induced apoptosis was demonstrated to be mediated partially through survivin downregulation, a member of IAPs family, in prostate cancer cells,^47^ the exact mechanisms have not yet been depicted. Our findings suggested that IAPs, to certain extent, might be pivotal regulators of BA‐induced apoptosis in HCC cells.

Autophagy is another major type of programmed cell death that has been identified to play an important role in cancer.^48^ Up to now, no studies have been reported to investigate the involvement of autophagy in BA‐induced cell death in HCC. Here, we showed that in the presence of chloroquine, BA‐induced cell death was attenuated, suggesting that BA induced HCC cell death via autophagy. Moreover, BA enhanced autophagic vacuole formation and accumulation, which further confirms the involvement of autophagy in HCC. Other studies have also confirmed that autophagy is involved in BA‐mediated cancer death.^49^, ^50^ BA induced cell death via reducing the overaccumulation of its protective autophagy through ubiquitin‐mediated degradation pathway in colorectal cancer cells,^49^ while it promoted cell death through the inhibition of autophagic flux in multiple myeloma cells.^50^ We also further studied the involvement of the autophagy pathway. Cells expressing mCherry‐GFP‐LC3 can be a powerful method to monitor autophagy flux.^37^ GFP‐LC3 and mCherry‐LC3 indicate autolysosome formation and degradation events, respectively. We noticed an increase in yellow puncta accumulation by BA, suggesting that BA inhibited autophagic flux in HCC cells. The conversion of LC3‐I to LC3‐II is used to monitor autophagy, and p62 is the most studied protein in the autophagy pathway. p62 binds to LC3 to be incorporated into the phagosomes, and they are degraded in autophagy process.^13^ If autophagy is induced, an increase in LC3‐II accumulation and p62 downregulation should be expected. However, it has been reported that LC3‐II accumulation does not always represent autophagy induction, and its accumulation may be due to the blockade of downstream degradation pathways.^51^ We showed that BA enhanced LC3‐1 conversion into LC3‐II and p62 accumulation, indicating that there is an accumulation of autophagosomes, without degradation of p62 and fusion with lysosomes, thus autophagic flux was inhibited. Therefore, we suggested that autophagic flux inhibition contributed to BA‐induced cell death in HCC. This is consistent with another study showing that BA induced cell death through the suppression of autophagic flux in microglia BV‐2 cells.^52^

Cell death is a complex process of physiological quality control, and different types of cell death may interplay or co‐exist. Several studies have showed the existence of the crosstalk between autophagy and apoptosis, two main types of programmed cell death.^53^ Although we observed that BA induced apoptosis and autophagy in HCC, the mechanisms behind the interaction of apoptosis and autophagy were not addressed. We postulated that IAPs could mediate the crosstalk between apoptosis and autophagy. The addition of autophagy and lysosomal inhibitors had no effect on IAPs expressions, so we concluded that BA induced apoptosis via autophagy‐independent IAPs degradation. Taken together, we suggest that apoptotic cell death through IAPs and autophagy contributed to BA‐induced cell death in HCC.

The oncogenic role of MALAT1 in tumorigenesis and progression of HCC has been recently suggested.^20^ It has also been reported to act as a proto‐oncogene modulating IAPs expression.^31^ Our investigation showed that BA inhibits IAPs expression and MALAT1 expression in HCC. Thus, we postulated that MALAT1 may take a functional role in BA‐induced apoptosis in HCC. MALAT1 acted as a sponge of miRNAs, leading to downregulation of miRNAs and subsequently abnormal expression of targeted genes.^25^, ^26^, ^27^ Here, we made a comprehensive search using the public database miRcode and Starbase, and miR‐22‐3p was identified to be a target gene of MALAT1, as well as an upstream gene of XIAP in cancer. Therefore, we hypothesized that MALAT1 may compete for miR‐22‐3p, attenuating the regulatory effect of miR‐22‐3p on IAPs. Our experiments demonstrated the apoptotic pathway of MALAT1/miR‐22‐3p/IAPs, and revealed that BA downregulated IAPs in HCC by targeting MALAT1 through the activation of miR‐22‐3p.

## 5 CONCLUSIONS

**FIGURE 7:**
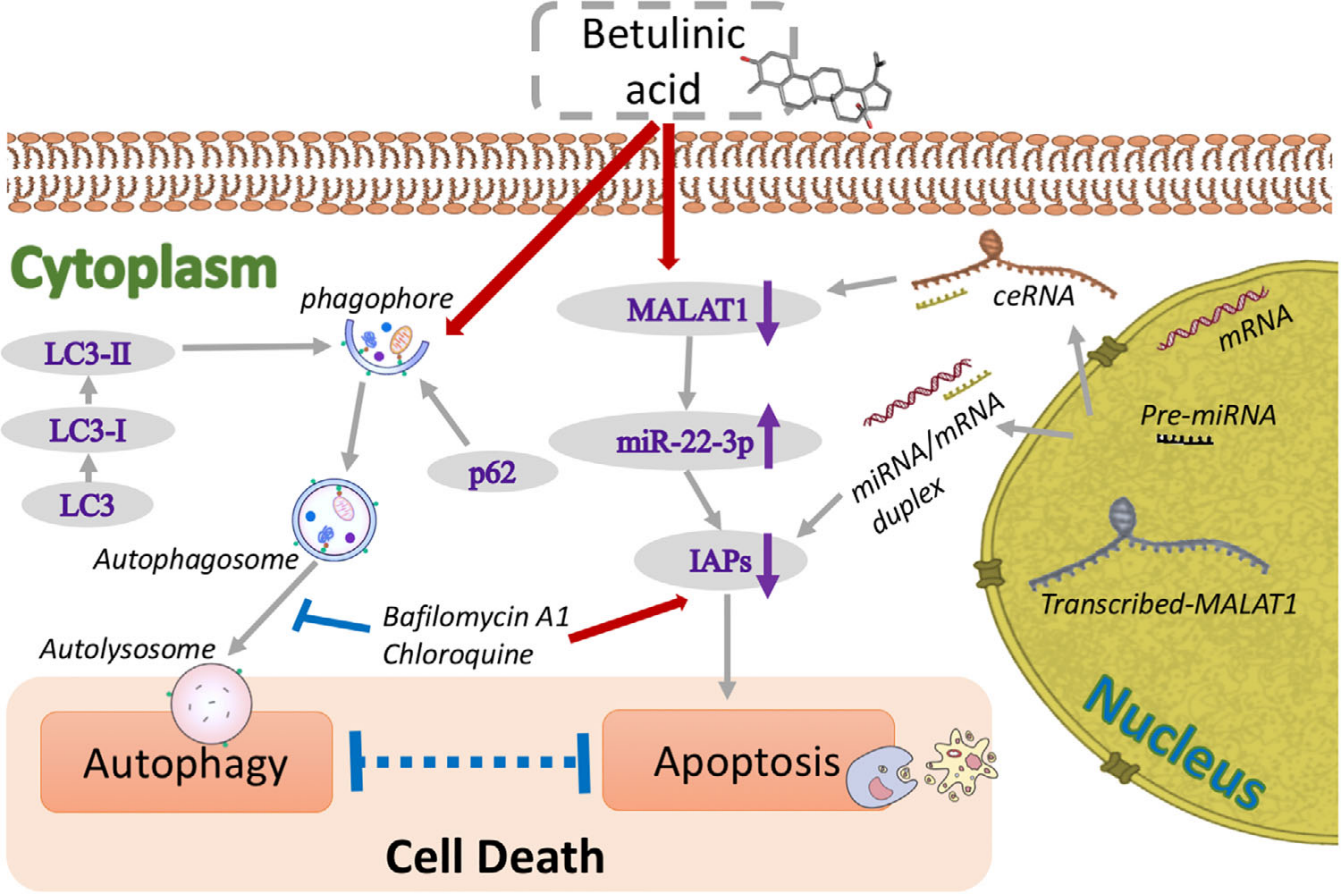
Diagram of the proposed mechanism underlying the inhibitory effects of BA on HCC. Exposure of BA induces cell death in HCC (1) through autophagic flux inhibition (2) and apoptosis via IAPs. The inhibition of autophagic flux is independent of IAPs pathway with the evidence that IAPs are not involved in BA‐mediated autophagic changes. Nevertheless, IAPs expressions decrease when the expression of proto‐oncogene MALAT1 is inhibited by BA, where MALAT1 functions as a ceRNA to contribute to BA‐induced apoptosis by sponging miR‐22‐3p

The mechanism through which BA induced cell death in HCC is presented in Figure 7. In summary, we demonstrated that BA, extracted from natural sources, exerted anticancer activity in HCC in vitro and in vivo, partly through autophagic flux inhibition and apoptosis via IAPs. The crosstalk between BA‐induced apoptosis and autophagy was independent of IAPs. Furthermore, we demonstrated that MALAT1 acted as a ceRNA of miR‐22‐3p, and BA affected the IAPs expression by targeting MALAT1 through the activation of miR‐22‐3p. Collectively, owing to its attractive property of being nontoxic to normal cells, BA may be a promising anticancer agent that regulates autophagy and apoptosis. Therefore, our study suggests that BA is a potential drug for HCC.

## CONFLICT OF INTEREST

The authors declare that there are no conflicts of interest.

## AUTHOR CONTRIBUTIONS

Yibin Feng and Junguo Ren conceived the idea, designed the study, and prepared the manuscript. Feiyu Chen did the experiments, analyzed the data, and drafted the manuscript. Zhangfeng Zhong and Ning Wang analyzed the data and commented the manuscript. Hor Yue Tan and Wei Guo revised the manuscript. Zhangfeng Zhong and Chien‐Shan Cheng polished the manuscript. All authors confirmed the final manuscript.

## Data Availability

The data that support the findings of this study are available on request from the corresponding author.
